# Iron-modified biochar improves plant physiology, soil nutritional status and mitigates Pb and Cd-hazard in wheat (*Triticum aestivum* L.)

**DOI:** 10.3389/fpls.2023.1221434

**Published:** 2023-08-17

**Authors:** Jari S. Algethami, Muhammad Kashif Irshad, Wasim Javed, Mohsen A. M. Alhamami, Muhammad Ibrahim

**Affiliations:** ^1^ Department of Chemistry, College of Science and Arts, Najran University, Najran, Saudi Arabia; ^2^ Promising Centre for Sensors and Electronic Devices (PCSED), Najran University, Najran, Saudi Arabia; ^3^ Department of Environmental and Energy Engineering, Yonsei University, Wonju, Republic of Korea; ^4^ Department of Environmental Sciences, Government College University Faisalabad, Faisalabad, Pakistan; ^5^ Punjab Bioenergy Institute (PBI), University of Agriculture Faisalabad, Faisalabad, Pakistan

**Keywords:** environmental pollution, iron modified biochar, remediation, soil enzymes, soil fertility

## Abstract

Environmental quality and food safety is threatened by contamination of lead (Pb) and cadmium (Cd) heavy metals in agricultural soils. Therefore, it is necessary to develop effective techniques for remediation of such soils. In this study, we prepared iron-modified biochar (Fe-BC) which combines the unique characteristics of pristine biochar (BC) and iron. The current study investigated the effect of pristine and iron modified biochar (Fe-BC) on the nutritional values of soil and on the reduction of Pb and Cd toxicity in wheat plants (*Triticum aestivum* L.). The findings of present study exhibited that 2% Fe-BC treatments significantly increased the dry weights of roots, shoots, husk and grains by 148.2, 53.2, 64.2 and 148%, respectively compared to control plants. The 2% Fe-BC treatment also enhanced photosynthesis rate, transpiration rate, stomatal conductance, intercellular CO_2_, chlorophyll a and b contents, by 43.2, 88.4, 24.9, 32.5, 21.4, and 26.7%, respectively. Moreover, 2% Fe-BC treatment suppressed the oxidative stress in wheat plants by increasing superoxide dismutase (SOD) and catalase (CAT) by 62.4 and 69.2%, respectively. The results showed that 2% Fe-BC treatment significantly lowered Cd levels in wheat roots, shoots, husk, and grains by 23.7, 44.5, 33.2, and 76.3%. Whereas, Pb concentrations in wheat roots, shoots, husk, and grains decreased by 46.4, 49.4, 53.6, and 68.3%, respectively. Post-harvest soil analysis showed that soil treatment with 2% Fe-BC increased soil urease, CAT and acid phosphatase enzyme activities by 48.4, 74.4 and 117.3%, respectively. Similarly, 2% Fe-BC treatment significantly improved nutrients availability in the soil as the available N, P, K, and Fe contents increased by 22, 25, 7.3, and 13.3%, respectively. Fe-BC is a viable solution for the remediation of hazardous Cd and Pb contaminated soils, and improvement of soil fertility status.

## 1 Introduction

Pollution of arable lands with injurious heavy metals (HMs), especially lead (Pb) and cadmium (Cd), and their accumulation in edible plant parts pose acute risks to human health. These metals have received increased attention due to their well-reported toxicity and widespread contamination of agricultural soils worldwide. Pb and Cd are non-essential HMs having no physiological or biochemical function in plants, but both can cause serious toxic effects on plants (Wang et al., 2020; Aqeel et al., 2021; Ran et al., 2023). The existence of high levels of Pb and Cd in plants inhibit root growth, reduce photosynthesis, increase oxidative stress, and limit other physiological activities of plants, ultimately resulting in reduced plant growth, yield, and quality (Islam et al., 2021; Aqeel et al., 2023; Yang et al., 2023). Moreover, long-term chronic exposure to toxic Cd and Pb causes a wide range of health hazards in human beings via the food chain (Guo and Li, 2019; He et al., 2021; Ajmal et al., 2022). Therefore, reclamation of polluted soils, along with reducing their uptake and accumulation in crop plants, is crucial for food safety.

Among various soil remediation techniques, *in-situ* immobilization of HMs in soil with organic materials such as biochar (BC) has been regarded a well-established, environmentally and economically viable remediation method (Beesley et al., 2011; Chen et al., 2018). BC is a carbon-rich material and reusing agricultural organic wastes and by-products as amendments in soil reclamation has recently gained tremendous popularity due to its cost-effective and environment friendly nature (Lehmann and Joseph, 2015). BC amendment increase soil organic matter, soil fertility and reduce soil pollution from pesticides and heavy metals due to its good adsorption abilities (Zhang et al., 2021; Chen et al., 2023). Nevertheless, the sorption of HMs, such as Cd and Pb, on BC surfaces is limited due to lesser specific surface area and fewer negative charges on biochar surfaces. Some previous studies have reported that applying pristine BC declines HMs toxicity in plants to some extent while improving overall soil health and plant growth (Wen et al., 2021). Various modification methods like loading mineral/metal salts on the BC surfaces can enhance BC’s HM adsorption capacity (Wang et al., 2020; Ali et al., 2022; Zhu et al., 2022). Modified biochar can efficiently limit the solubility and bioavailability of HMs in soil by affecting metals speciation, absorption, precipitation, and complexation mechanisms (Wang et al., 2020; Chandra et al., 2023). In accordance with the concept of sustainable development, it is crucial to prepare modified biochar as an efficient, stable, economical, and environment-friendly amendment for the reclamation of HMs polluted soils.

A few studies have suggested that biochar-loaded Fe materials *i.e*., zero-valent Fe nano-particles, Fe-oxides, and Fe-sulfides, may be useful to reduce HMs uptake in plants (Guo and Li, 2019; Wan et al., 2020; Wen et al., 2021). There is evidence that Fe-oxides could reduce the solubility of Cd, Pb, and other HMs in soil, which in turn could alleviate their phytoavailability and leaching potential (Wang et al., 2020; Irshad et al., 2022a). Minerals such as Fe-oxides play a key role in soil not only as nutrient sources for plants but also as aggregators and sorbents of HMs. Iron-modified biochar (Fe-BC) can adequately immobilize HMs due to its larger surface area, microporous structure, and active functional groups. Several studies illustrated that Fe-BC enhanced Cd and Pb immobilization by up to 60% and reduced their bioavailability (Xu et al., 2016; Wan et al., 2020; Yang et al., 2021b). Although Fe-BC has the potential to immobilize Cd and Pb, its effect on soil remediation is not fully understood.

In most parts of the world, wheat (*Triticum aesitvum* L.) is an important staple food. Wheat grown in Cd and Pb-polluted soil accumulates more Cd and Pb mainly through roots and translocate to aerial parts, eventually accumulating in wheat grains. Cd and Pb ingestion from such polluted crops is considered one of the significant dietary sources of health risks, including cancer, gastrointestinal problems, etc. Keeping in view the above-mentioned facts current study involves the development of sustainable method for reclamation of Cd and Pb from soil-wheat systems with an emphasis on improving soil and plant health. It is hypothesized that Fe-BC, which combines the unique characteristics of both BC and Fe-oxide, may improve its sorption capacity of multi-metals from soil. Specifically, the current investigation focused (i) to determine the effect of Fe-BC on wheat growth and physiology, (ii) to assess the potential of Fe-BC for phytoavailability and immobilization of Cd/Pb, (iii) to assess the efficacy of Fe-BC in reclaiming HMs polluted soil along with a focus on soil fertility improvement.

## 2 Materials and methods

This experiment was conducted using analytical grade reagents procured from Sigma-Aldrich, USA. Corncob feedstock used for the preparation of BC was collected from a clean field.

### 2.1 Biochar characterization

Biochar was developed by pyrolysis of corncob at 600 °C under limited oxygen supply conditions. To prepare Fe-BC the corncob BC was submerged in FeCl_3_·6H_2_O solution at 20:1 (biochar: Fe *w/w*). In short, the BC and Fe solution were stirred for 20 minutes followed by rotary shaking for 1 hour at 25 °C. The mixture was heated for 1 hour at 600 °C (Dong et al., 2016). After drying, Fe-BC was passed through 2 mm sieves and stored in sealed containers. We recorded FTIR (Fourier Transform Infrared Spectra) with a Bruker Vector 22 FTIR spectrometer in the 4000–400 cm^-1^ region. A Brunauer-Emmett-Teller surface area measurement was performed. We determined the crystal structure of the adsorbents using XRD (Bruker D2 Phaser) with CuK radiation (= 1.5406 Å).

After digestion with HNO_3_ and HClO_4_, Cd, Pb and Fe content in both biochars was calculated using atomic absorption spectrometer (SP-IAA4530, China) and inductively coupled plasma mass spectrometry (Nex ION 1000 ICP MS), respectively. The physicochemical properties of biochar, Fe-modified biochar and soil are presented in **Table 1**.

**Table 1:** Physicochemical properties of biochar, iron modified biochar and soil.

Characteristics	Unit	Biochar	Iron modified biochar	Soil
pH		7.4 ± 0.2	7.2 ± 0.1	5.5 ± 0.1
EC	(dS m^-1^)	2.4 ± 0.3	3.8 ± 0.2	3.7 ± 0.3
Fe	(g kg^-1^)	0.75 ± 0.14	21.5 ± 0.22	1.21 ± 0.15
BET Surface area	m^2^ g	57	210	ND
Total Pb	(mg kg^-1^)	ND	ND	445 ± 5.2
Total Cd	(mg kg^-1^)	ND	ND	4.2 ± 0.34
C	(%)	54.1 ± 0.3	49.4 ± 0.4	ND
N	(%)	2.9 ± 0.2	3.2 ± 0.1	ND
P	(%)	1.55 ± 0.1	1.7 ± 0.1	ND
K	(%)	2.80 ± 0.1	2.94 ± 0.1	ND
Available P	(mg kg^-1^)	ND	ND	5.2 ± 0.09
Available N	(mg kg^-1^)	ND	ND	175.2 ± 4.3
Extractable K	(mg kg^-1^)	ND	ND	130 ± 1.4
Texture: Clay loam				
Sand	(%)			38
Silt	(%)			32
Clay	(%)			30

ND, not detected.

### 2.2 Soil collection and characterization

Soil (0-20 cm) was sampled from farmland near Faisalabad, Pakistan. Air-dried soil was sieved through 2 mm sieves. The soil was artificially spiked at 500 mg Pb kg^-1^ soil and 10 mg Cd kg^-1^ soil, using lead nitrate (Pb(NO_3_)_2_) and cadmium chloride (CdCl_2_) solutions, respectively (Xu et al., 2023). In 1:5 soil-to-water (*w/v*) suspension, the pH and EC of the soil were determined using a pH-EC meter. Soil texture was determined by hydrometer method (Bouyoucos, 1962). The Cd and Pb contents were measured by atomic absorption spectrometer (SP-IAA4530, China) (Bai et al., 2023).

### 2.3 Wheat growth experiment

In December 2022, a pot experiment was carried out at Government College University Faisalabad, Pakistan. Briefly, BC and Fe-BC were mixed with polluted soil at 1% BC, 2% BC, 3% BC, 1% Fe-BC, 1.5% Fe-BC, and 2% Fe-BC (*w/w* basis) and thoroughly homogenized. A control group (CK) without BC was also included. A completely randomized design, having four replicates was employed. Every plastic pot was filled up with 4 kg of polluted soil. After surface sterilization with sodium hypochlorite (NaClO) wheat (Var. Punjab-2011) seeds were rinsed with hydrogen peroxide (H_2_O_2_) and deionized water. Four wheat plants were grown in each pot. Upon completion of 120 days of growth, wheat plants were harvested. The shoot length was determined at the time of harvesting. Root length was calculated with Win RHIZO. The plant parts were placed in an oven at 65°C for 72 hours and dry weights were determined.

### 2.4 Photosynthesis measurement

Leaf samples collected at 60 days of growth were used for the extraction of chlorophyll contents by using acetone (85% *v/v*). Samples were placed at 4°C in dark conditions until all chlorophyll content was extracted from the leaves. Based on the absorbance of the samples at specific wavelengths (663 and 647 nm), the chlorophyll content was calculated using a spectrometer (Lamdbda 365 UV-Vis spectrometer, Perkin Elmer). Chl a and Chl b values were calculated following (Lichtenthaler, 1987).


Chl a = (12.25 × A663) – (2.79×A647)

Chl b = (21.5 × A647) – (5.1×A663)

Stomata conductance, photosynthetic rate, intercellular CO_2_, and transpiration rate were recorded with an Infra-Red Gas Analyzer (IRGA LI6400/XT, Germany).

### 2.5 Determination of plant oxidative stress and enzyme activities

For electrolyte leakage measurement (EL) the leaf samples were chopped and extracted at 32°C for 120 minutes in water bath to obtain the initial electrical conductivity (EC1) of the solution, followed by further extraction for 20 minutes at 121°C to determine the final electrical conductivity (EC2). EL was calculated following (Dionisio-Sese and Tobita, 1998).


EL (%) = (EC1/EC2) ×100

As a measure of lipid peroxidation, malondialdehyde (MDA) was calculated following (Zhang and Kirkham, 1994). The MDA was determined at wavelengths 532 and 610 nm using a spectrophotometer (Lamdbda 365 UV-Vis spectrometer, Perkin Elmer). Whereas, hydrogen peroxide (H_2_O_2_) concentrations were measured following (Jana and Choudhuri, 1982). Activities of superoxide dismutase (SOD) and peroxidase (POD) were determined at 560, 470 nm absorbance by following the protocols developed by (Zhang, 1992). Whereas, CAT activity was determined at 240 nm absorbance by following (Aebi, 1984; Chen et al., 2022).

### 2.6 Wheat Cd and Pb content measurement

After harvest, plant parts were digested with HNO_3_ and HClO_4_. The Cd and Pb in samples were measured by an atomic absorption spectrometer (SP-IAA4530, China).

### 2.7 Determination of soil enzyme activities, DTPA extractable Cd, Pb and nutrients

Soil was collected and stored at -20°C after harvesting. To assess the effectiveness of applied amendments, soil catalase (CAT), acid phosphatase and urease were determined. We determined the activities of urease and CAT enzyme in soil following (Mahamood et al., 2023). Acid phosphatase was measured following (Tabatabai and Bremner, 1969). We extracted the plant-available Cd and Pb from the soil using AB-DTPA extraction (pH 7.3) (Martens and Lindsay, 1990). A pH meter was used to measure the soil pH after harvesting. Available phosphorus (P) and potassium (K) were extracted with 0.5 mol L^-1^ NaHCO_3_ and 1 mol L^-1^ NH_4_AC solutions. P and K were measured by using atomic absorption spectrometer (SP-IAA4530, China) and an automatic intermittent chemical analyzer (Smartchem 200, Italy), respectively. We determined nitrogen (N) availability using the alkali diffusion method (Wang et al., 2023). Soil Fe content was determined following Irshad et al. (2022a).

### 2.8 Statistical analysis

STATISTIX version 8.2 software was used for statistical analyses. We tested differences between treatments using analysis of variance (ANOVA) at 0.05 significance levels. The experiment was laid out in completely randomized design (CRD) with four replicates. All data reported are means of four replicates. The correlation matrix and Principal Component Analysis (PCA) were executed and developed by using R-studio v. 4.0.5.

## 3 Results and discussion

### 3.1 Characterization of biochar

**Figure 1:**
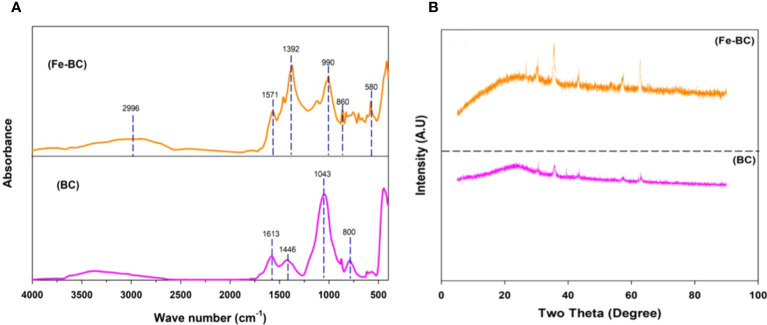
FTIR spectra **(A)** and XRD pattern **(B)** of biochar (BC) and iron-modified biochar (Fe-BC).

FTIR spectrometry confirmed the existence of different functional groups on Fe-BC surfaces (**Figure 1A**). The broad peak at 2996 cm^-1^ reveals the presence of stretching vibrations of -OH/Fe–OH, possibly caused by iron coating on the biochar surface (Chen et al., 2011). The 1571 cm^-1^ peak shows COO- or C=C groups. Moreover, we observed 1392 cm^-1^ vibrations of CH_2_ units associated with C-H deformation. Whereas, FTIR spectrum of Fe-BC shows a band of 580 cm^-1^ corresponding to iron oxide signals (Lim et al., 2009; Irshad et al., 2020). In BC, the peak at 1613 cm^-1^ reveals aromatic C=C and C=O and the peak at 1043 demonstrates a carbon-oxygen bond (C-O-C). Whereas a peak at 1446 cm^-1^ donates CH_2_ (Uchimiya et al., 2010). XRD patterns also showed the presence of iron on biochar surfaces (**Figure 1B**). It is interesting to note that the new peaks at 30.4 cm^-1^ are strongly associated with the presence of iron in Fe-BC (Wang et al., 2015). Further, the peaks at 43.7 cm^-1^ indicate an increase in iron oxide particles. An iron peak can be observed at 57.9 cm^-1^ and 62.8 cm^-1^ in Fe-BC (Korobeinyk et al., 2011).

### 3.2 Impact of BC and Fe-BC applications on wheat biomass

**Figure 2:**
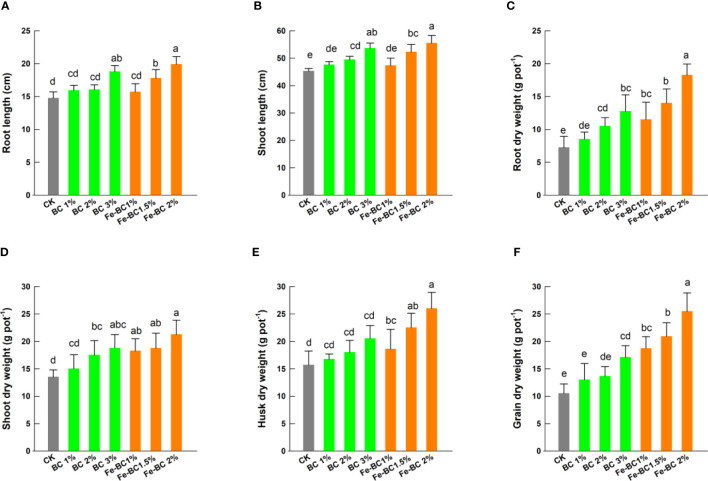
Effect of biochar (BC) and iron-modified biochar (Fe-BC) on root length **(A)**, shoot length **(B)**, root dry weights **(C)**, shoot dry weights **(D)**, husk dry weights **(E)** and grain dry weights **(F)** of wheat plants. Different letters on the bars show significant differences at (*p< 0.05*). Values are means ± SD (n=4).

Under different treatments, both amendments significantly affected the plant growth in terms of plant height, root length, roots, shoots, husk, and grains dry weight (*p<0.05*) (**Figure 2**). The root and shoot lengths increased in the following ordered: CK >1% BC >2% BC >3% BC >1% Fe-BC >1.5% Fe-BC >2% Fe-BC. Soil supplementation with BC 3% treatment enhanced the wheat roots, shoots, husk and grains dry weight by 68.5, 34.8, 28.4 and 137.2%, respectively. Soil application of 2% Fe-BC resulted in the largest increase in plant biomass. Results exhibited that dry weights of roots, shoots, husk and grains increased by 148.2, 53.2, 64.2 and 148%, respectively, when 2% Fe-BC was applied. Fe-BC amendments also improved soil available N, P, K and Fe contents, which may have promoted the wheat plant growth (**Table 2**). The increased nutrient availability and improved soil enzyme activities after adding BC could have contributed to the increased plant dry weights. A decrease in oxidative damage is related to an increase in plant growth and biomass under Cd and Pb toxic soils. Our observations are in accordance with (Li et al., 2021; Mahamood et al., 2023) that nZVI and biochar composite application have positive impacts on plant biomass.

**Table 2:** Effect of biochar (BC) and iron-modified biochar (Fe-BC) on soil properties, soil nutrients and DTPA extractable Cd and Pb.

Treatments	pH	Available P	Available N	Extractable K	Fe	Soil AB-DTPA extractable Cd	Soil AB-DTPA extractable Pb
		(mg kg^-1^)					
CK	5.5 ± 0.1e	5.2 ± 0.09e	175.2 ± 4.3 f	130 ± 1.4 d	1.21 ± 0.05 f	1.41 ± 0.09 a	297.2 ± 2.7 a
BC 1%	5.6 ± 0.1de	5.3 ± 0.1de	182 ± 1.63 d	132 ± 2.2 cd	1.3 ± 0.04 ef	1.31 ± 0.1 a	264.5 ± 3.65 b
BC 2%	5.7 ± 0.2cd	5.7 ± 0.09 c	189 ± 2.4 e	134 ± 1.8 bc	1.47 ± 0.06 de	1.11 ± 0.08 a	240.4 ± 1.82 c
BC 3%	5.9 ± 0.1b	6.1 ± 0.06 b	195 ± 3.7 c	136 ± 2.7 ab	1.57 ± 0.1 d	0.85 ± 0.09 c	211.5 ± 3.26 d
Fe-BC 1%	5.6 ± 0.2de	5.4 ± 0.14 d	191 ± 1.9 cd	131.2 ± 4.2 cd	2.3 ± 0.12 c	0.9 ± 0.14 c	245.3 ± 4.08 e
Fe-BC 1.5%	5.8 ± 0.1bc	5.8 ± 0.09 c	203 ± 2.5 b	133.5 ± 2.4 bcd	2.7 ± 0.1 b	0.67 ± 0.06 d	221.1 ± 2.44 f
Fe-BC 2%	6 ± 0.1a	6.5 ± 0.12 a	215 ± 4.1 a	139.2 ± 2.9 a	3.6 ± 0.09 a	0.4 ± 0.09 e	184.7 ± 4.1g

Values are means ± SD (n=4). Different letters indicate significant differences at p ≤ 0.05.

### 3.3 Plant photosynthesis

**Figure 3:**
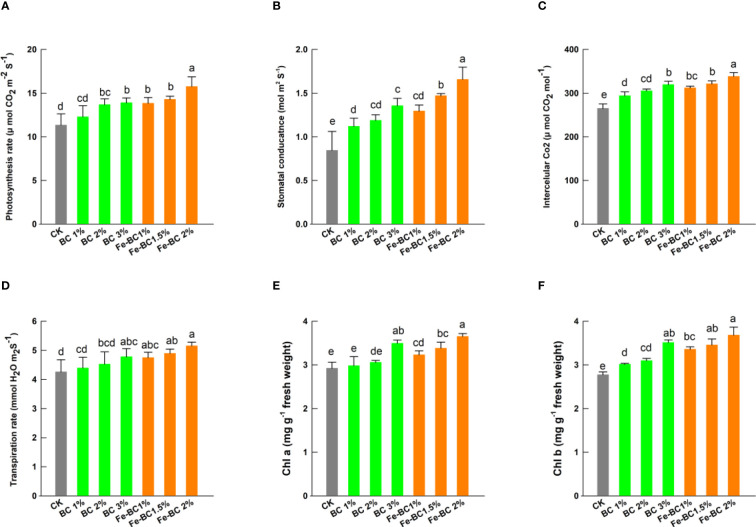
Effect of biochar (BC) and iron-modified biochar (Fe-BC) on photosynthesis rate **(A)**, stomatal conductance **(B)**, intercellular CO_2_ **(C)**, transpiration rates **(D)**, chlorophyll a **(E)** and chlorophyll b contents **(F)** of wheat plants. Different letters on the bars show significant differences at (*p< 0.05*). Values are means ± SD (n=4).

Photosynthesis and chlorophyll contents increased significantly in plants treated with BC and Fe-BC (**Figure 3**). The soil addition of 3% BC treatment augmented the chlorophyll a and b contents by 16.4 and 21.5%, respectively, over the control. However, Fe-BC increased the chlorophyll a and chlorophyll b content by 21.4 and 26.7%, respectively. The photosynthetic rate was significantly increased by both treatments. It was noted that 3% BC treated plants showed an increase in photosynthesis by 25.4% in comparison with the non-treated plants. In contrast, 2% Fe-BC increased the photosynthetic rate by 43.2%. As compared to control, 3% BC amendment increased the respiration rate by 14.4%, while 2% Fe-BC application enhanced the respiration rate by 24.9%. A significant enhancement in stomatal conductance was also noted in both amendments. The 2% Fe-BC exhibited the highest stomatal conductance (88.4%). A similar trend in the increase of intercellular CO_2_ was observed with both soil amendments. The 2% Fe-BC produced the highest intercellular CO_2_ among all applied treatments (32.5%). Plants require chlorophyll (Chl a, Chl b) for the production of food through photosynthesis. Cd accumulates in plants and inhibits the uptake of nutrients that are necessary for chlorophyll synthesis (Hu et al., 2019). Increased plant defence and decline in oxidative stress might explain the improvement in chlorophyll content. Both BC and Fe-BC increased chlorophyll a and b contents linearly with increasing application rate, suggesting that they protected chlorophyll against Cd and Pb damage. Because of chlorophyll’s high content, photosynthesis is more efficient (Ghassemi-Golezani et al., 2020). Moreover, soil amendment with BC and Fe-BC has declined the Cd and Pb uptake and increased chlorophyll contents along with augmented photosynthetic attributes (Ajmal et al., 2022).

### 3.4 Plant oxidative stress and enzyme activities

**Figure 4:**
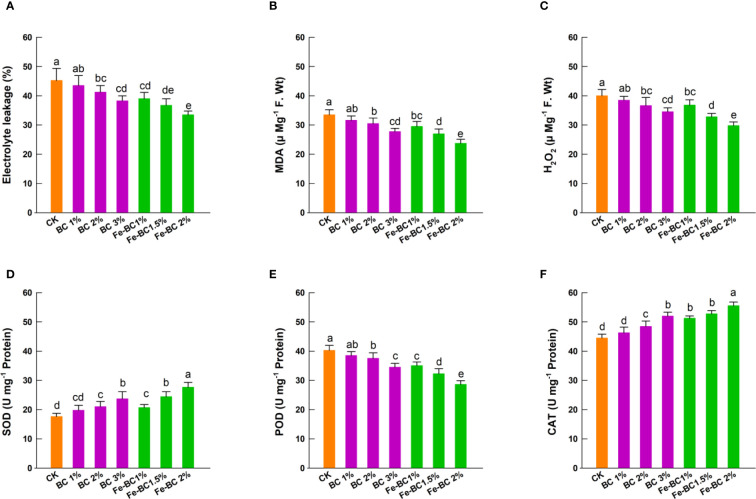
Effect of biochar (BC) and iron-modified biochar (Fe-BC) on EL **(A)**, MDA **(B)**, H_2_O_2_ **(C)**, SOD **(D)**, POD **(E)**, and CAT **(F)** of wheat leaves. Different letters on the bars show significant differences at (*p< 0.05*). Values are means ± SD (n=4).

When plants are under HMs stress, they exhibit oxidative stress (EL, H_2_O_2_ MDA, etc.). We examined different antioxidant enzymes to determine ability of BC and Fe-BC to control Cd and Pb-induced oxidative damage. **Figure 4** illustrates the changes in H_2_O_2_, EL, MDA, and antioxidants (SOD, CAT, and POD) in the leaf tissues of wheat plants. In comparison to the control, pristine BC and Fe-BC reduced H_2_O_2_, EL, and MDA contents in plants significantly. In control, the highest values of H_2_O_2_, EL, and MDA were obtained, while the lowest values were obtained with 2% Fe-BC treatment.

The results exhibited that 3% BC treatment resulted in a 17.3, 13.2, and 14.4% decrease in H_2_O_2_, EL, and MDA contents, respectively. However, 2% Fe-BC reduced H_2_O_2_, EL, and MDA contents in wheat leaves by 29.3, 33, and 30%, respectively, compared to controls. As indicated by the results, the Cd and Pb stress negatively affected SOD, POD, and CAT activities in the control plants. SOD and CAT activity correlated positively with BC and Fe-BC ratios (**Figures 4D, F**). As compared with the control, 3% BC amendment increased SOD and CAT activity by 38.4 and 22.1%, respectively. Results indicated that a maximum increase in SOD and CAT activity was observed with plants amended with 2% Fe-BC. In comparison with the control, 2% Fe-BC amendment increased SOD and CAT activity by 62.4 and 29.4%, respectively. A 34.5% decrease in POD activity was observed after 2% Fe-BC application (**Figure 4E**). The membranes of plants that are under Cd or Pb stress produce huge amount of reactive oxygen species (ROS). These ROS are peroxidised by the fatty acids in the membranes. According to the current study, plants grown in BC and Fe-BC treated soils experience less oxidative stress due to lower levels of Cd and Pb. The BC application also decreased the build-up of EL, H_2_O_2_ and MDA (Farhangi-Abriz and Torabian, 2017). An earlier study reported that BC could significantly reduce oxidative stress in radish, amaranth, and soybean plants grown on As, Pb, Cd, Cu, and Zn contaminated soils (Jiang et al., 2022). The antioxidant enzyme activities of plants represent their ability to counter the oxidative stress caused by HMs. After Fe-BC addition, wheat plants might have experienced less oxidative stress due to increased antioxidant enzyme activities (Xu et al., 2020).

### 3.5 Cadmium and lead content in plant tissues

**Figure 5:**
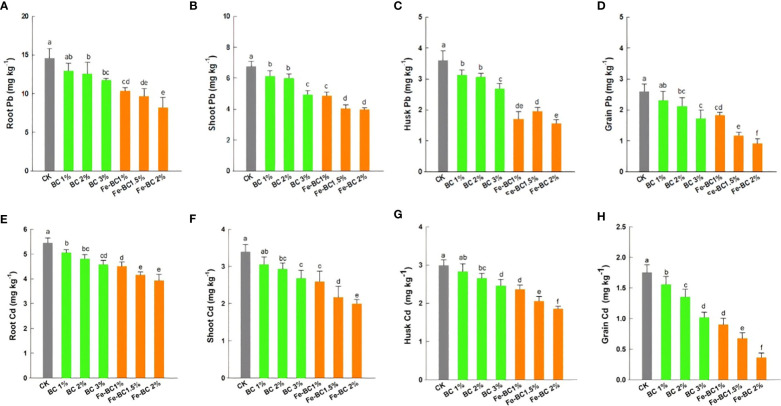
Effect of biochar (BC) and iron-modified biochar (Fe-BC) on root lead **(A)**, shoot lead **(B)**, husk lead **(C)**, grain lead **(D)**, root cadmium **(E)** shoot cadmium **(F)** husk cadmium **(G)** and grain cadmium **(H)** content of wheat plants. Different letters on the bars show significant differences at (*p< 0.05*). Values are means ± SD (n=4).

Our findings exhibited that BC and Fe-BC significantly decreased the concentrations of Cd and Pb in wheat plant parts (*p< 0.05*) (**Figure 5**). The Cd and Pb concentration was highest in control plants tissues, while Fe-BC treated plants had the lowest concentration. A decrease in Pb content in wheat tissues was noted in the following orders: CK >1% BC >2% BC >3% BC >1% Fe-BC >1.5% Fe-BC >2% Fe-BC. As compared to control soil, 3% BC and 2% Fe-BC amended soils decreased Pb concentrations in wheat roots by 22.2 and 46.4%, respectively. Similarly, Pb concentrations in wheat shoots decreased by 28.4 and 49.4%, respectively, with 3% BC and 2% Fe-BC treatments, respectively. The maximum reduction in Pb concentration (53.6%) in husk and grains (68.3%) was observed with 2% Fe-BC treatment. Wheat plants showed a similar decline in Cd uptake in response to both amendments. When compared with BC and control, 2% Fe-BC treatments showed the greatest decline in Cd uptake in the wheat plant. When 2% Fe-BC was added to the soil, Cd concertation in wheat roots, shoots, husk, and grains decreased by 23.7, 44.5, 33.2, and 76.3%, respectively. The decrease in Cd and Pb phytoavailability observed in BC and Fe-BC treated soils may be attributed to an increase in soil pH (Palansooriya et al., 2020). Moreover, decrease in Pb and Cd bioavailability in Fe-BC amended soil is caused due to augmented soil Fe, surface complexation/precipitation of Pb and Cd to Fe oxides (Li et al., 2019; Irshad et al., 2022b). Fe-BC soil application also increased the availability of P thus causing the development of Cd_3_ (PO_4_)_2_ and Cd-Cd phosphate, which ultimately decreases its mobility in soil and decline its uptake in plants tissues (**Table 2**) (Yang et al., 2021b). In addition, transformations and speciation of iron might have altered clay mineral properties, affecting the phytoavailability and mobility of Cd and Pb, and thus interfering with their plant absorption (Yu et al., 2020). The increased BET surface area of modified biochar may have facilitated the Cd and Pb adsorption and limited Cd and Pb absorption in wheat (Zhang et al., 2020).

### 3.6 Soil enzyme activities

**Figure 6:**
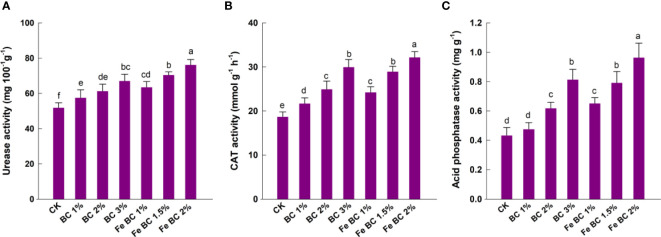
Effect of biochar (BC) and iron-modified biochar (Fe-BC) on soil urease **(A)**, catalase **(B)**, and acid phosphatase **(C)** activities. Different letters on the bars show significant differences at (*p< 0.05*). Values are means ± SD (n=4).

Soil enzyme activity is closely related to soil nutrient cycling as affected by soil organic amendments. In the soil nitrogen cycle, urease converts nitrogen into small molecule forms that can be directly used by plants and microbes. Acid phosphates catalyzes phosphate and anhydrides to enhance phosphorus utilization (Wang et al., 2023). In the present study, soil amendments positively correlated with soil enzyme activity. It was found that the soil enzyme activity was highest when Fe-BC was applied at its maximum rate. The 2% Fe-BC increased the urease activity by more than double, whereas 3% BC treatment increased it by only 23.7% (**Figure 6**). A similar increase in CAT activity and acid phosphatase activity was observed with Fe-BC amendments. Soil supplementation with 2% Fe-BC significantly enhanced the CAT and acid phosphatase activities by 74.4 and 117.3%, respectively, in comparison with the control. CAT activity of soil is highly dependent on pH, and Fe-BC addition increased the soil pH, which lead to an increase in CAT activity (Liu et al., 2020). BC has a porous structure, that facilitates microorganism growth and improves soil quality, texture, and nutrient contents (Song et al., 2022). A decrease in metal bioavailability and toxicity could also lead to an increase in CAT and urease activities in Fe-BC-treated soils (Irshad et al., 2022a). We confirmed earlier findings that iron-modified biochar improved soil enzyme activity in Cd, Pb, and As-polluted paddy soils (Wen et al., 2021).

### 3.7 Effect of biochar on soil nutrients, DTPA Extractable Cd and Pb

The nutrient contents of soil significantly improved with both amendments (**Table 2**). Soil application of 2% Fe-BC significantly increased available N, P, K and Fe content by 22, 25, 7.3 and 13.3%, respectively. Biochar amendment has been reported as a promising and sustainable method to improve soil quality and fertility over the past few decades (Gao et al., 2022; Rasse et al., 2022). Our results are in accordance with earlier studies that soil application of biochar increased the supply of nutrients to the soil and thereby improved the plant growth (Lin et al., 2023; Wang et al., 2023). A linear decrease in soil bioavailable Cd and Pb was observed with increasing application rates of both amendments. The maximum decline in bioavailable Cd and Pb was noted with 2% Fe-BC amendment (**Table 2**). Elevated soil pH values after Fe-BC addition might have increased the Cd and Pb immobilization (Wang et al., 2018). According to several previous studies, soil supplementation with iron-treated biochar minimized Cd and Pb bioavailability due to an increased supply of Fe (Yin et al., 2017; Rajendran et al., 2019). It has been observed that biochar and Fe-modified biochar addition to the soil has positively resulted in the increase in available N and available P and thus making good impact on the growth of the plants.

### 3.8 Correlation analysis

**Figure 7:**
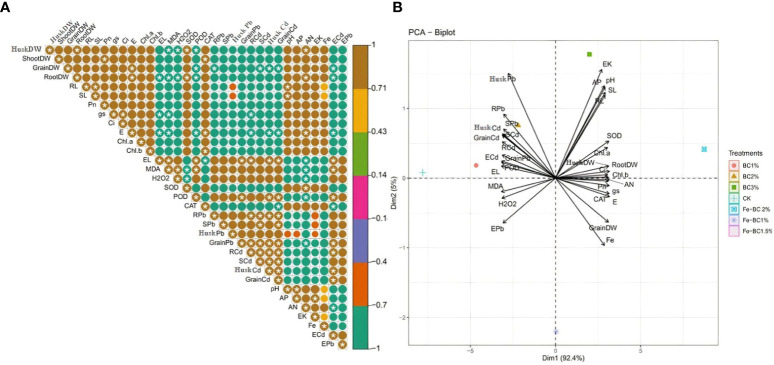
The correlation matrix reveals the effects of BC, Fe-BC, Cd and Pb on plant growth, physiology, metal concentration in plant tissues and soil nutrient status. Dark browns and green show significant positive and significant negative correlations, * shows significance at p ≤ 0.05 **(A)**. Principal component analysis of wheat conducted with and without Cd and Pb stress. A PCA-biplot illustrates interaction among various attributes of wheat plants amended with BC and Fe-BC **(B)**.

The interaction between the various investigated attributes was analysed using Pearson correlation and principal component analysis (**Figure 7**). Morphological attributes such as dry weights of husk, shoot and grains, length of root and shoot, and photosynthetic traits showed a marked positive association with each other. Likewise, antioxidant contents also demonstrated positive interactions with each other and with other lead and cadmium contents, while electrolyte leakage has a significantly positive interaction (*p* ≤ 0.05) with plant cadmium contents as well with MDA and POD but showed significantly negative correlation (*p* ≤ 0.05) with CAT. These HMs, which had accumulated in various plant parts, hurt both the morphology and the photosynthetic apparatus of plants. Our correlation also demonstrated that metal accumulation in plants is associated with increased hydrogen peroxide and electrolyte leakage of the plant tissues. These findings are following the observation of Mahamood et al., 2023, Chen et al. (2022); Aqeel et al. (2021), which showed that HMs such as Pb and Cd had a significant negative impact on the biomass of plants as well as photosynthetic efficiency. The correlation also showed that soil nutrient levels were elevated when both biochar were applied to the soil. There was a positive correlation between increased soil available N, P, K, and Fe with plant growth and photosynthesis of wheat plants. The development of principal component analysis has further validated our correlation matrix results. It also demonstrated that photosynthetic characteristics and plant morphological traits are closely related to each other, while MDA, POD, H_2_O_2_, EL as well as Pb and Cd contents have close relation whit one another. Moreover, different dimensions of PCA synergistically accounted for about 98% of the data set’s variability, with Dim-1 and Dim-2 each accounting for 94.1% and 3.4% of the overall variation, respectively.

Most of the studied attributes accounted mainly on Dim-1 as explained by the percentage variability. Our PCA result also indicated the plant morphological and photosynthetic attributes grouped together which show they have close relationship with each other and behaves similar to the applied treatments. In contrast, antioxidants, Pb concentrations, and EL had radically different findings and interacted with each other’s.

## 4 Conclusions

The current study involves combining the benefits of iron and biochar to prepare iron-modified biochar (Fe-BC). The potential impact of BC and Fe-BC on the soil mobility and bioaccumulation of Cd and Pb in wheat grains were quantitatively assessed. Our findings demonstrated an efficient reduction in Cd and Pb bioavailability when Fe-BC was applied to polluted soil. Soil application of Fe-BC also increased the amount of available nutrients to the plants and boosted the biomass of wheat roots, shoots, husk, and grains. Supplementing the soil with 2% Fe-BC significantly reduced metal-induced oxidative stress in wheat plants. The soil application of 2% Fe-BC significantly reduced Cd and Pb accumulation in wheat. Fe-BC supply to soil also improved soil health in terms of soil enzymes. It is concluded that Fe-BC has the potential to remediate Cd and Pb polluted soils and limit their uptake in wheat plants. Moreover, Fe-BC is capable of serving as an alternative to conventional synthetic soil fertilizers.

## Data availability statement

The raw data supporting the conclusions of this article will be made available by the authors, without undue reservation.

## Author contributions

JA and MKI performed the experiment and write up the paper draft. WJ, MI and MA helped in conducting of experiment and paper revision. MI provided the guidance to carry out the experiment and revised the draft. All authors contributed to the article and approved the submitted version.
